# Improved phylogenetic resolution for Y-chromosome Haplogroup O2a1c-002611

**DOI:** 10.1038/s41598-017-01340-z

**Published:** 2017-04-25

**Authors:** Xiaotian Yao, Senwei Tang, Beilei Bian, Xiaoli Wu, Gang Chen, Chuan-Chao Wang

**Affiliations:** 1 WeGene, Shenzhen, 518040 China; 2 School of Information Science and Engineering, Central South University, Changsha, 410083 China; 3 Department of Archaeogenetics and Eurasia3angle research group, Max Planck Institute for the Science of Human History, D-07745 Jena, Germany; 4 Department of Genetics, Harvard Medical School, Boston, MA 02115 United States

## Abstract

Y-chromosome Haplogroup O2a1c-002611 is one of the dominant lineages of East Asians and Southeast Asians. However, its internal phylogeny remains insufficiently investigated. In this study, we genotyped 89 new highly informative single nucleotide polymorphisms (SNPs) in 305 individuals with Haplogroup O2a1c-002611 identified from 2139 Han Chinese males. Two major branches were identified, O2a1c1-F18 and O2a1c2-L133.2 and the first was further divided into two main subclades, O2a1c1a-F11 and O2a1c1b-F449, accounting for 11.13% and 2.20% of Han Chinese, respectively. In Haplogroup O2a1c1a-F11, we also determined seven sublineages with quite different frequency distributions in Han Chinese ranging from 0.187% to 3.553%, implying they might have different demographic history. The reconstructed haplogroup tree for all the major clades within Haplogroup O2a1c-002611 permits better resolution of male lineages in population studies of East Asia and Southeast Asia. The dataset generated in the present study are also valuable for forensic identification and paternity tests in China.

## Introduction

The phylogeny of Y-chromosome provides a powerful tool to reconstruct genetic relationship of human populations and paternal lineages^1–3^. Haplogroup O-M175 is a dominant component of the East Asian Y-chromosome gene pool, accounting for 75% of the total paternal lineages of Chinese^4–9^. Haplogroup O-M175 gave rise to two main downstream haplogroups-O1-M265 and O2-M122 - totaling 60% of the Y chromosomes among East Asian populations^4–9^. The Haplogroup O1a-M119, a sublineage of O1-M265, is prevalent along the southeast coast of China, occurring at high frequencies in Tai-Kadai speaking and Taiwan Austronesian-speaking people^8, 9^. Another sublineage of O1, O1b-M268, accounts for about 5% of the Han Chinese^4^. The most frequent subclade of O1b is O1b1a1a-M95, which is the dominant haplogroup in the Indo-China Peninsula and is suggested to be associated with Austroasiatic speaking people^8, 9^. Another subclade of O1b, O1b2-M176, is particularly enriched in Koreans and Japanese and could be probably associated with Yayoi people who brought agriculture to Japan and Korea^10, 11^. The O2-M122 is the most common lineage in China and is also prevalent throughout surrounding regions, comprising roughly 50 to 60% of the Han Chinese^4–9^. There are three main subclades of O2-M122, called O2a1c-002611, O2a2b1-M134 and O2a2b1a1-M117, with each accounting for 12 to 17% of the Han Chinese^4–9^. The O2a2b1a1-M117 also reaches high frequencies in Tibeto- Burman speaking populations in southwest China^9^. The Haplogroup O2a1c-002611 is also prevalent in different ethnic groups in East Asia and Southeast Asia, comparing 14% of Vietnamese, and about 5% of Manchu and Mongol^12, 13^. The Y-STR diversity shows a general south-to-north decline of Haplogroup O2a1c-002611, which is consistent with the prehistorically northward migration of the other O2-M122 lineages^12^.

The importance of O2a1c-002611, aside from its genetic prevalence, is its distinctive role together with other O2 lineages in the formation of the Sino-Tibetan language family, the second largest family in the world in terms of population size. There are two main sublineages in Haplogroup O2a1c-002611 defined by two single nucleotide polymorphisms (SNPs) F11 and F238, respectively^12^. The lineage O2a1c1a-F11 is suggested to be one of the three super-grandfathers for present-day Chinese that experienced star-like expansions in Neolithic Era at about 6 kya (thousand years ago)^14^. The frequencies of Haplogroup O2a1c-002611 and its sublineages are relatively low in Tibeto-Burman speaking populations (0–3%), which suggests the lineage expansions in ancient Han Chinese might begin immediately after the separation of the ancestors of the Han Chinese and Tibeto-Burman^12, 15, 16^. The Haplogroup O2a1c-002611 probably didn’t participate in the formation of Tibeto-Burman groups but was heavily involved in the origin and expansion of Han Chinese^12, 15, 16^.

Despite its abundance, wide distribution and the importance to Sino-Tibetan populations, the phylogeny of Haplogroup O2a1c-002611 has not been adequately resolved with respect to O-M95^17^ and O-M134^18^. The population history of Han Chinese remains unclear because the phylogeny of Haplogroup O2a1c-002611 still lacks resolution with no downstream markers having been genotyped and described in large scale sample collections and the phylogenetic positions of those markers having yet to be determined. To date, the only two markers investigated in literature internal to O2a1c-002611 have been F11 and F238^12^, which were not sufficient to resolve the phylogeny of the lineages belonging to this haplogroup. The recent next-generation sequencing of East Asian samples has yielded a variety of novel SNPs purportedly belonging to the O2a1c-002611 lineage^14, 19–21^. Here, we describe a large-scale, nationwide study of Haplogroup O2a1c-002611 in Han Chinese by using high-density genotype data to examine phylogenetic positions of newly reported markers and provide useful tools for future population history analysis.

## Methods

All participants were drawn from the customer base of WeGene, Inc., a consumer personal genetics company. The study was conducted in accordance with the human and ethical research principles of The Ministry of Science and Technology of the People’s Republic of China (Interim Measures for the Administration of Human Genetic Resources, June 10, 1998). Participants provided informed consent and participated in the research online, under a protocol approved by the Ethical Committee of WeGene, Inc.

DNA extraction and genotyping were performed on saliva samples. Samples have been genotyped on WeGene V1 genotyping platform using Affymetrix arrays with a total of about 596,000 SNPs. Quality control (QC) was performed in PLINK V1.07^22^. The individuals and SNPs with genotype call rate of <98.5% were excluded. The relatedness was checked pair wisely for all the samples and where identity by descent (IBD) scores of >0.125 (3rd-degree relative) were identified with one from each such pair removed. The individuals whose analyses failed repeatedly were recontacted by WeGene customer service to provide additional samples, as is done for all WeGene customers. The WeGene V1 arrays were designed to identify all known Y-chromosome lineages with 18963 Y-chromosome phylogenetic relevant SNPs. In this study, we investigated 89 SNPs that overlap with the markers listed in ISOGG O2a1c-002611 phylogenetic tree accessed on 21 April 2016, with 14 August 2016 correction (http://www.isogg.org/). Here, we follow the regulations proposed by the Y Chromosome Consortium^23^ which defined a set of rules about how to update the haplogroup names and phylogenetic trees of Y-chromosome.

## Results

**Figure 1:**
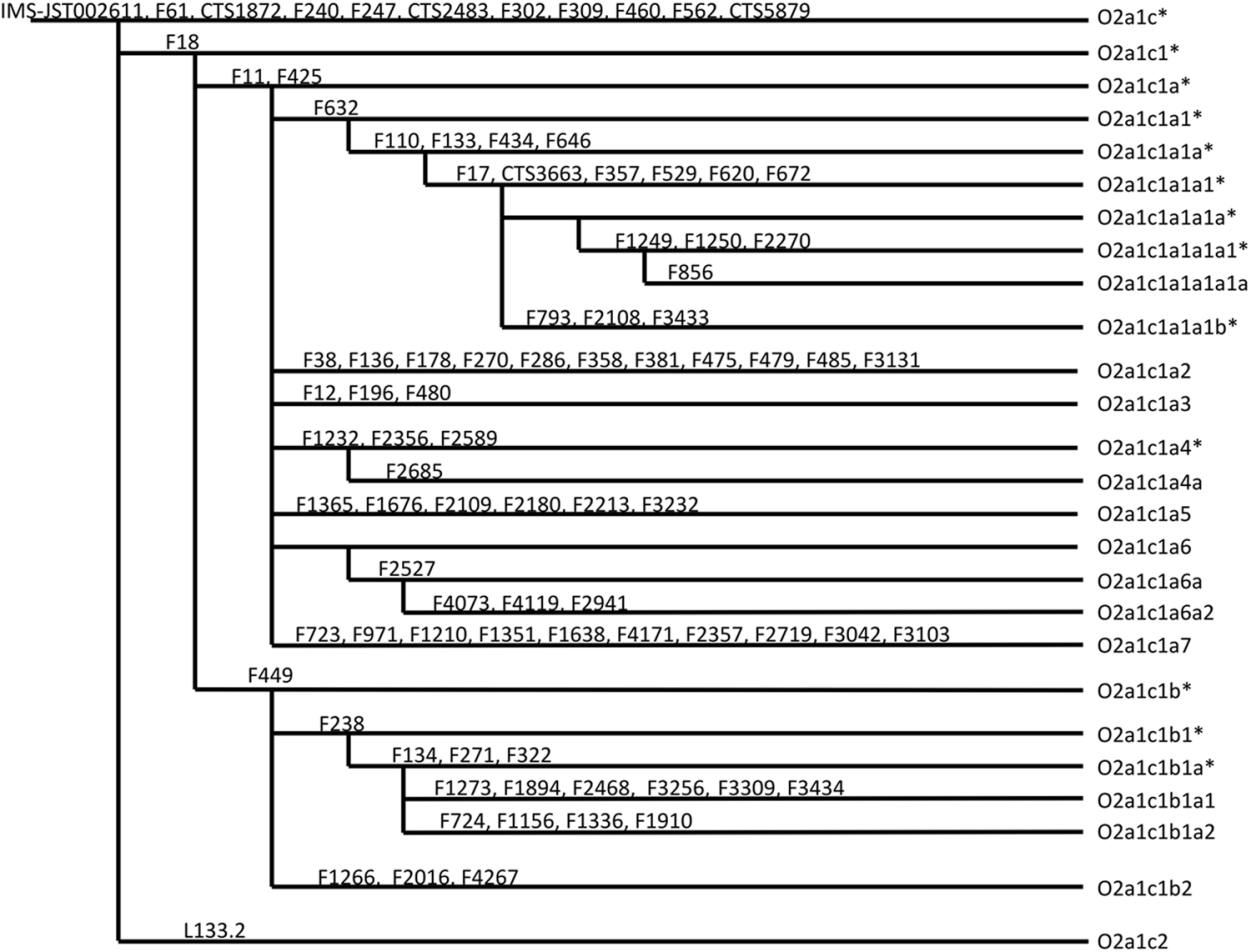
Updated phylogenetic tree of the human Y-chromosome lineage O2a1c-002611.

Among the 2139 male individuals, 305 of them (14.26%) belong to the O2a1c-002611 lineage (Table 1), in agreement with previous studies of East Asian populations^4, 12–14^. For these individuals with a derived allele at IMS-JST002611, we investigated other 88 SNPs purportedly belonging to the O2a1c-002611 haplogroup (genotyping results with hg19 physical positions and sample locations are given in Table S1), and the results allowed us to update the phylogenetic tree of O2a1c-002611. We applied the parsimony rule in tree construction. For example, F61, CTS1872, F240, F247, CTS2483, F302, F309, CTS5879, F460, and F562 showed derived status in all IMS-JST002611 derived samples, supporting that they are equivalent with IMS-JST002611 in the phylogeny. For F18, the majority samples have derived alleles, but we did find some showing ancestral status, indicating that F18 is a downstream SNP of IMS-JST002611 (Fig. 1).

**Table 1:** The frequencies of Haplogroup O2a1c-002611 in Han Chinese.

Haplogroup	Count	Frequency			
		Sum	East	North	South
O2a1c*	1	0.05%	0.05%	0.00%	0.00%
O2a1c1*	12	0.56%	0.09%	0.14%	0.33%
O2a1c1a*	53	2.48%	0.94%	0.33%	1.22%
O2a1c1a1*	8	0.37%	0.19%	0.00%	0.19%
O2a1c1a1a*	27	1.26%	0.37%	0.56%	0.33%
O2a1c1a1a1*	14	0.66%	0.23%	0.09%	0.33%
O2a1c1a1a1a1*	1	0.05%	0.00%	0.00%	0.05%
O2a1c1a1a1a1a	6	0.28%	0.14%	0.00%	0.14%
O2a1c1a1a1b	20	0.94%	0.19%	0.51%	0.23%
O2a1c1a2	7	0.33%	0.00%	0.33%	0.00%
O2a1c1a3	16	0.75%	0.37%	0.23%	0.14%
O2a1c1a4*	6	0.28%	0.05%	0.19%	0.05%
O2a1c1a4a	4	0.19%	0.05%	0.05%	0.09%
O2a1c1a5	57	2.67%	0.79%	1.17%	0.70%
O2a1c1a6a	6	0.28%	0.05%	0.05%	0.19%
O2a1c1a6a2	9	0.42%	0.28%	0.09%	0.05%
O2a1c1a7	4	0.19%	0.05%	0.05%	0.09%
O2a1c1b1*	13	0.61%	0.05%	0.28%	0.28%
O2a1c1b1a*	9	0.42%	0.09%	0.09%	0.23%
O2a1c1b1a1	17	0.80%	0.19%	0.56%	0.05%
O2a1c1b1a2	1	0.05%	0.05%	0.00%	0.00%
O2a1c1b2	7	0.33%	0.09%	0.19%	0.05%
O2a1c2	7	0.33%	0.05%	0.14%	0.14%
total	305	14.26%	4.35%	5.05%	4.87%

The “East” refers to the samples whose origins are from the provinces of Jiangsu, Anhui, Zhejiang, and Shanghai, whereas ‘North’ and ‘South’ refers to the other provinces of which the capitals locate northern or southern to the Line of Qinling Mountains-Huai River, respectively.

We identified two sub-branches within Haplogroup O2a1c-002611: O2a1c1-F18 and O2a1c2-O2a1c2. The previously genotyped F11^12^ is suggested to be a downstream marker of F18. The O2a1c1-F18 is the main subclade, accounting for 97.38% of all the O2a1c-002611 samples. The Haplogroup O2a1c1-F18 is further divided into two main subclades, O2a1c1a-F11 (the other equivalent SNP is F425) and O2a1c1b-F449, accounting for 11.13% and 2.20% of the Han Chinese, respectively. The subclade O2a1c1a-F11 was further split into seven sub-branches, named O2a1c1a1-F632, O2a1c1a2-F38 (other equivalent SNPs are F136, F178, F270, F286, F358, F381, F475, F479, F485, and F3131), O2a1c1a3-F12 (other equivalent SNPs are F196 and F480), O2a1c1a4-F1232 (other equivalent SNPs are F2356 and F2589), O2a1c1a5-F1365 (other equivalent SNPs are F1676, F2109, F2180, F2213, and F3232), O2a1c1a6 (here we didn’t type the determined SNP listed on ISOGG for this lineage, but we have downstream markers that identify the subclade O2a1c1a6a-F2527 and O2a1c1a6a2-F4073, F4119, F2941), and O2a1c1a7-F723 (other equivalent SNPs are F971, F1210, F1351, F1638, F4171, F2357, F2719, F3042, and F3103). The previously genotyped F238^12^ is suggested to be a downstream marker of F449. The other subclade of O2a1c1b-F449 is O2a1c1b2-F1266 (the other two equivalent SNPs are F2016 and F4267).

Our identification of the seven branches within O2a1c1a-F11 is consistent with the previous finding^14^ that this lineage probably experienced huge population expansion in Neolithic Time. However, those seven sub-branches show quite different frequency distributions in Han Chinese ranging from 0.187% in O2a1c1a7 to 3.553% in O2a1c1a1. The frequency of O2a1c1a5 in Han Chinese also reaches 2.665%, while the frequencies of other four sub-branches are all below 1% (Table 1).

The geographic distribution pattern of Haplogroup O2a1c-002611 in our current study is consistent with previous estimations that this haplogroup enriches in the eastern part of China. The population in Jiangsu, Anhui, Zhejiang, and Shanghai have nearly one-third of the males belonging to this lineage as shown in Table 1. There are interesting substructures in distributions regarding different sublineages. One of the two main subclades of O2a1c-002611, O2a1c1a-F11 (and its sublineages), is equally distributed in eastern, northern and southern China regarding frequency. However, the other subclade O2a1c1b-F449 and its sublineages O2a1c1b1-F238 and O2a1c1b2-F1266 are particularly enriched in northern China with a frequency of 1.12% but only 0.47% and 0.61% in eastern and southern China, respectively. The observation is consistent with our hypothesis in Wang *et al*.^12^ that mutation of O2a1c1b1-F238 probably occurred in Proto-Han-Chinese in northern China after the split with Tibeto-Burman and other southern native populations. The lineage O2a1c1a*-F11 (the samples only have derived alleles at sites F11 and F425 but other no downstream derived SNPs) is two to three times lower in frequency in northern China compared with that in eastern and southern China, and we have not found O2a1c1a1*-F632 in northern China. However, Haplogroup O2a1c1a1a1b, O2a1c1a5, O2a1c1b1a1, and O2a1c1b2 are more frequent seen in northern China than in southern and eastern China.

## Discussion

Haplogroup O2a1c-002611 is frequently distributed in East Asia and surrounding areas. The genotyping of 89 phylogenetic relevant SNPs under Haplogroup O2a1c-002611 enables us to refine and update the phylogeny of this lineage. The reconstructed haplogroup tree for all the major clades within Haplogroup O2a1c-002611 permits better resolution of male lineages in population studies of East Asia and surrounding areas.

This study shows that the 89 SNPs are highly informative for separating a substantial part of O2a1c-002611 samples in China. We observe a huge expanded lineage named O2a1c1a-F11 within Haplogroup O2a1c-002611, comprising 11.13% of the Han Chinese. There are seven subclades nested within O2a1c1a-F11, suggesting the expansion of this lineage is star-like^7^. Those subclades might have experienced different demographic histories since they were separated from a common ancestor because the frequencies of those subclades in present-day Han Chinese are so different ranging from 0.187% to 3.553%. A similar pattern has been observed in another Neolithic expanded lineage O-F46. There are two subclades O-F209 and O-F2887 under O-F46 that reach high frequencies in Han Chinese (~3% and ~4.2%, respectively), while the other four subclades O*-F46, O-F48, O-F3386, O-F1739 are not frequent or even extremely rare^11^. One possible explanation for this uneven expansion is a social selection that a few paternal lineages achieved a greater continuous advantage on the existing basis of the early expanded farming population that enabled them to have more decedents.

Since the Haplogroup O2a1c-002611 has distinct distributions in Han Chinese and Tibeto-Burman populations and probably experienced agriculture-induced expansion, exploring the detailed phylogenetic relationships of the subclades in this lineage is not only informative for tracing prehistoric migrations, but also for understanding the origin and diversification of Sino-Tibetan language family in the future. For instance, although Haplogroup O2a1c-002611 is rare in Tibeto-Burman groups, we have found it at 1% to 3% in Qiangic speaking populations, such as Muya, Jiarong, Queyu and Qiang in the Tibeto-Burman Corridor^12^. The Qiangic speaking groups are suggested to have played an important role in the formation of Sino-Tibetan populations based on historical documents, linguistics, and genetic studies^15, 24, 25^. To genotype the Qiangic speaking populations with this improved phylogeny of Haplogroup O2a1c-002611 will certainly provide detailed information in understanding the origin of Sino-Tibetans.

We note a limitation of our study is that we have only genotyped Haplogroup O2a1c-002611 in Han Chinese samples, but this haplogroup has also been found with moderate or even high frequency in various ethnic groups in southern China, Laos, Vietnam, and Philippines^12, 13, 26^. Detailed characterization of this haplogroup could provide a broader framework of peopling East Asia and Southeast Asia.

The recent next-generation sequencing of worldwide samples has yielded tens of thousands of novel SNPs on Y chromosome purportedly being phylogenetic relevant^14, 19–21^. But it is extremely time and money consuming (or even impossible) to validate all those markers by the PCR and SNaPshot techniques that we usually used in the previous studies^4, 8, 9, 12, 15^. Here, we give a successful example of how the consumer-based genetic test with the advent of microarray SNP genotyping technology could be used in Y-chromosome phylogeny analysis. The reconstructed phylogeny of these new markers in this study is only the first step, and the real benefit will come from typing a large number of O2a1c-002611 derived individuals of various phylogeographic and ethnic backgrounds, which will certainly broad our understanding of the population history.

## Electronic supplementary material

Table S1
